# Both Underweight and Obesity Are Associated With an Increased Risk of Coronavirus Disease 2019 (COVID-19) Severity

**DOI:** 10.3389/fnut.2021.649422

**Published:** 2021-10-08

**Authors:** Pian Ye, Ran Pang, Ling Li, Hua-Rong Li, Shuang-Lin Liu, Lei Zhao

**Affiliations:** ^1^Department of Infectious Diseases, Tongji Medical College, Union Hospital, Huazhong University of Science and Technology, Wuhan, China; ^2^Department of Integrated Traditional Chinese and Western Medicine, Tongji Medical College, Union Hospital, Huazhong University of Science and Technology, Wuhan, China; ^3^Department of Urology, Tongji Medical College, Wuhan No. 1 Hospital, Huazhong University of Science and Technology, Wuhan, China

**Keywords:** COVID-19, BMI, SARS-CoV-2, underweight, obesity, acute lung injury

## Abstract

**Introduction:** As coronavirus Disease 2019 (COVID-19) has evolved into a global pandemic, increasing numbers of reports have linked obesity to more severe COVID-19 illness and death. However, almost all the studies focused on an increased risk of mortality or intensive care unit (ICU) admission among hospitalized obese patients with COVID-19. Is obesity also associated with the incidence of acute lung injury (ALI) in the patients with COVID-19? How about underweight patients? The answer is lacking. Therefore, our following research will answer the above two questions.

**Methods:** We collected and analyzed epidemiologic, demographic, clinical, and laboratory data from 193 confirmed cases of COVID-19 at Union Hospital, Tongji Medical College, Huazhong University of Science and Technology in Wuhan, China, between January 1, 2020, and March 13, 2020. They were followed up until April 15, 2020. Underweight was defined by body mass index (BMI) lower than 18.5 kg/m^2^, normal weight by 18.5−23.9 kg/m^2^, overweight by 24.0−27.9 kg/m^2^, and obesity as ≥28 kg/m^2^.

**Results:** Among these patients, 5.70% were underweight, 58.03% were normal weight, 27.98% were overweight, and 8.29% were obese. Underweight patients were more likely to have a headache (*P* = 0.029). Obese patients were more likely than other groups to experience a decline in lymphocyte counts (*P* = 0.038), an increase in C-reactive protein (CRP; *P* = 0.023), bilateral multiple mottling, and ground glass opacity in the lungs (*P* = 0.007). Besides, the proportion of patients receiving human immunoglobulin + systematic corticosteroids treatment is the highest among the obese group compared with other BMI groups. After adjusting for potential confounders, underweight patients had a 6.483-fold higher (*P* = 0.012), and obese patients showed a 5.965-fold higher odds for developing ALI than normal-weight patients (*P* = 0.022). In addition, underweight patients were 3.255 times more likely than normal-weight patients to develop secondary infections (*P* = 0.041).

**Conclusions:** Our study showed that both underweight and obese patients with COVID-19 tend to develop ALI compared with normal-weight patients. Underweight patients were more likely to develop a secondary infection than other patients.

## Introduction

Since December 2019, unexplained pneumonia cases have been found in the hospitals in Wuhan, Hubei province, China. These patients were then confirmed to be infected by a novel coronavirus called severe acute respiratory syndrome coronavirus 2 (SARS-CoV-2). Up to now, SARS-CoV-2 has swept the world, with outbreaks occurring in more than 200 countries and regions around the world and covering seven continents (including Antarctica). According to official statistics, more than 2,290 million patients have been confirmed and more than 4,700,000 patients have died, with a mortality rate of about 2.05%. Although many people have been known to be infected with SARS-CoV-2, some characteristics of COVID-19 and how they affect disease progression are not fully understood. Previous studies have found that comorbidities, such as hypertension, diabetes, and cardiovascular disease, are associated with a more severe disease course of COVID-19. Several subsequent studies have shown that obesity was emerging as a risk factor for susceptibility and severity of COVID-19 (1–4). “Obese people are significantly more likely to become seriously ill and be admitted to ICU with COVID-19 compared to those with a healthy BMI” (5). However, almost all studies focused on the proportion of obese patients admitted to the ICU or an increased risk of mortality in obese patients. Is obesity also correlated with the incidence of ALI in patients with COVID-19? How about underweight patients? Previous studies did not tell us the answer. So, our research focuses on answering the above questions.

## Materials and Methods

### Study Design and Participants

For this single-center retrospective cohort study, all confirmed adult cases with COVID-19 having standing body height and weight drawn on the day of admission to the general isolation ward of Union Hospital, Tongji Medical College, Huazhong University of Science and Technology, Wuhan (also known as Wuhan Union Hospital) between January 1, 2020, and March 15, 2020, were enrolled. To avoid influencing BMI determination, four pregnant women were excluded. They were followed up until April 15, 2020. All patients met the diagnostic criteria set out in the WHO guidelines for COVID-19. This study was approved by the ethics committee of Wuhan Union Hospital and obtained oral consent from all the patients because the exemption from written consent of informed patients was approved by the ethics committee owing to the rapid spread of the infection and the rapid progression of some cases. Founded in 1866, Wuhan Union Hospital is a major general hospital engaged in medical treatment, medical research, and education. It is directly under the administration of the National Health Commission. The hospital is only 4 km away from the Huanan Seafood Wholesale Market where the COVID-19 outbreak first started and was responsible for the diagnosis and treatment of suspected cases during the early stage of the COVID-19 outbreak.

### Inclusion Criteria

The main inclusion criteria were the following:

(1) Adult patients (age ≥ 18 years) presenting with fever and/or active respiratory symptoms related to SARS-CoV-2 infection were admitted to the general isolation ward between January 1, 2020, and March 15, 2020.
(2) Confirmed case of COVID-19 by PCR.
(3) Patients having standing body height and weight drawn on the day of admission.

To minimize confounding factors that may affect our evaluation of the effect of BMI on the severity of COVID-19, we excluded four pregnant female patients since the normal range of BMI in pregnant women is different from that of the general population, and pregnancy may have some unknown effects on severity of COVID-19.

### Procedures

On admission, anthropometric measures, including standing body height and weight of all subjects, were recorded wearing light indoor clothing but without shoes. Height was measured to the nearest 0.1 cm, and weight was recorded to the nearest 0.1 kg. BMI was calculated as the ratio of weight (kilograms) divided by height (meters) squared (kg/m^2^). Chinese-specific four categories of BMI cutoffs were used, lower than 18.5 kg/m^2^ is defined as underweight, 18.5–23.9 kg/m^2^ is defined as normal weight, 24.0–27.9 kg/m^2^ is defined as overweight, and ≥28.0 kg/m^2^ is defined as obesity. Then we collected epidemiologic, demographic, present history, suspicious contact history, comorbidities, symptoms, signs, laboratory tests, imaging, and treatment programs from the electronic medical records (EMR) in the general isolation ward of Wuhan Union Hospital. The presence of COVID-19 was confirmed by real-time PCR (RT-PCR) assays were approved by the State Food and Drug Administration, using the same protocol as previously described (6). RT-PCR detection reagents were provided by institutions. All the tests were conducted in the clinical laboratory department of Wuhan Union Hospital. All patients underwent several lung CT scans. Treatment options include respiratory support, antiviral therapy, antibiotics, human immunoglobulin, and corticosteroid therapy. Repeated tests for SARS-CoV-2 and pulmonary CT reexamination were performed in patients with confirmed SARS-CoV-2 infection. Two consecutive negative results with quantitative PCR (q-PCR) detection over an interval of 24 h were performed to verify that the virus was completely cleared before discharge or isolation was terminated. If the information in the EMR is incomplete or needs to be verified, we obtained the exact information through direct communication with attending doctors, other healthcare professionals, or patients themselves. All data were checked by two physicians.

Disease onset was defined as the time when the first symptom was noticed. Since only two of these patients died, one was transferred to the ICU ward due to the aggravation of pneumonia and finally died in ICU. Another patient died in the general isolation ward. We judged the severity of pneumonia of the patient according to whether they have ALI or acute respiratory distress syndrome (ARDS).

### Definitions

ALI and ARDS were defined according to the Berlin definition (7). Secondary infection was diagnosed if the patients had clinical symptoms or signs of nosocomial pneumonia or bacteremia and was combined with a positive culture of a new pathogen from a lower respiratory tract specimen (including the sputum, transtracheal aspirates, or bronchoalveolar lavage fluid, or from blood samples taken ≥48 h).

### Statistical Analysis

For categorical variables, we calculated the frequency rates and percentages of patients in each category. Proportions for categorical variables were compared using the χ^2^ test, although the Fisher's exact test was used when the data were limited. We summarized continuous variables as either means and SD or medians with interquartile ranges. Medians for continuous variables were compared using the independent group *t*-tests when the data were normally distributed; otherwise, the independent sample Kruskal-Wallis test was used. Multivariable logistic regression was used to analyze the risk factors for ALI and secondary infection. Odds ratios (ORs) and 95% CIs were calculated. A two-sided α of < 0.05 was considered statistically significant. We used SPSS (version 24.0) for all analyses.

## Results

By March 15, 2020, all the 193 patients with confirmed COVID-19 were included in this study. They were mainly infected by patients or colleagues in hospitals, by family members at home, or by others without their knowledge outside the home. Each of them tested positive for SARS-CoV-2 at least once before admission. And the images of the lung CT of the patients show signs typical of viral pneumonia. Table 1 shows that most of the infected patients are women [122 (63.21%)].

**Table 1:** Characteristics of 193 patients with COVID-19.

	**Total (***n*** = 193)**	**BMI, Kg/m** ^2^				***P*** **-value**
		**Normal (***n*** = 112, 58.03%)**	**Underweight (***n*** = 11, 5.70%)**	**Overweight** **(***n*** = 54, 27.98%)**	**Obesity (***n*** = 16, 8.29%)**	
Age, year, Median (IQR)	39 (32–56.5)	37.5 (31–54.75)	36 (31-61)	40.5 (33.75–57.25)	44 (38-55)	0.236
Males, N (%)	71/193 (36.79)	32/112 (28.57)	1/11 (9.09)	27/54 (50)	11/16 (68.75)	< 0.001
**Personal coexisting chronic disease, N (%)**						
Hypertension	54/193 (27.98)	22/112 (19.64)	2/11 (18.18)	20/54 (37.04)	10/16 (62.50)	< 0.001
Malignant tumor	18/193 (9.33)	11/112 (9.82)	0/11 (0)	5/54 (9.26)	2/16 (12.5)	0.824
Diabetes	17/193 (8.81)	9/112 (8.04)	0/11 (0)	8/54 (14.81)	0/16 (0)	0.236
Cardiovascular disease	57/193 (29.53)	24/112 (21.43)	2/11 (18.18)	21/54 (38.87)	10/16 (62.50)	0.002
**Initial symptoms, N (%)**						
Fever	160/193 (82.90)	89/112 (79.46)	9/11 (81.82)	48/54 (88.89)	14/16 (87.50)	0.491
Cough	121/193 (62.69)	70/112 (62.50)	7/11 (63.64)	35/54 (64.81)	9/16 (56.25)	0.145
Fatigue	94/193 (48.70)	48/112 (42.86)	4/11 (36.36)	32/54 (59.26)	10/16 (62.50)	0.122
Headache	24/193 (12.44)	13/112 (11.61)	4/11 (36.36)	4/54 (7.41)	3/16 (18.75)	0.029
Diarrhea	32/193 (16.58)	20/112 (17.86)	2/11 (18.18)	8/54 (14.81)	2/16 (12.5)	0.932
Sore throat	29/193 (15.03)	17/112 (15.18)	4/11 (36.36)	6/54 (11.11)	2/16 (12.5)	0.224
Shortness of breath	63/193 (32.64)	38/112 (33.93)	3/11 (27.27)	15/54 (27.78)	7/16 (43.75)	0.632
**Disease progression, days, Median (IQR)**						
From onset to hospitalization	7 (5-12)	8 (6-14)	5 (3-15)	7 (4.75–10.25)	7 (4.25–8.75)	0.291
From onset to dyspnea	5.5 (3–7.25)	6 (4.5–8)	6 (1)	5 (2-6)	4 (0–8)	0.120
From onset to ARDS	8 (7-11)	11 (7-29)	-	9 (5.5–10)	7 (7-7)	0.500
From onset to discharge	25 (20-34)	26 (21–37.5)	31 (21-43)	23 (17.75–28)	22 (19.25–30)	0.072
**Auxiliary examination results**						
Lymphocytes below normal (< 1.1 G/L), N (%)	70/193 (36.27)	45/112 (40.18)	4/11 (36.36)	12/54 (22.22)	9/16 (56.25)	0.038
Albumin below normal(< 35 g/L), N (%)	27/193 (13.99)	16/112 (14.29)	2/11 (18.18)	6/54 (11.11)	3/16 (18.75)	0.726
Prealbumin below normal (<0.17 g/L), N (%)	72/180 (40)	39/102 (38.24)	6/10 (60)	19/52 (36.54)	8/16 (50)	0.421
CRP above normal (≥8 mg/L), N (%)	98/192 (51.04)	52/111 (46.85)	2/11 (18.18)	34/54 (62.96)	10/16 (62.50)	0.023
**lung CT scans**						
Bilateral multiple mottling and groundglass opacity, N (%)	93/193 (48.19)	49/112 (43.75)	4/11 (36.36)	26 (48.15)	14/16 (87.50)	0.007
**Severity of COVID-19**						
Acute lung injury, N (%)	96/193 (49.74)	48/112 (42.86)	8/11 (72.73)	26 (48.15)	14/16 (87.50)	0.003
ARDS, N (%)	18/193 (9.33)	9/112 (8.18)	1/11 (9.09)	5 (9.26)	3/16 (18.75)	0.478
Secondary infection, N (%)	30/193 (15.54)	13/112 (11.61)	4/11 (36.36)	10 (18.52)	3/16 (18.75)	0.116
**Treatment**						
Combination of antibiotics were administered after admission, N (%)	101/193 (52.33)	54/112 (48.21)	8/11 (72.73)	28 (51.85)	11/16 (68.75)	0.234
Human immunoglobulin+systematic corticosteroids, N (%)	49/193 (25.39)	21/112 (18.75)	2/11 (18.18)	19 (35.19)	7/16 (43.75)	0.036
oxygen therapy, N (%)	143/193 (74.09)	78/112 (69.64)	9/11 (81.82)	42 (77.78)	14/16 (87.5)	0.343

*BMI, body mass index; ARDS, acute respiratory distress syndrome*.

Among all these patients, 5.70% were underweight, 58.03% were normal weight, 27.98% were overweight, and 8.29% were obese. The proportion of men in each group increased with the increase in BMI, they were 9.09, 28.57, 50, and 68.75%, respectively (*P* < 0.001). Patients with underweight are more likely to have a headache (*P* = 0.029). Obese patients have a greater chance of developing some personal coexisting chronic diseases, such as high blood pressure (*P* < 0.001) and cardiovascular diseases (*P* = 0.002). Besides, in the obese group, more patients have decreased lymphocyte count and increased CRP levels compared with other groups (*P* = 0.038, *P* = 0.023; out of these patients, one lacked CRP count). Intriguingly, Even though underweight patients were the least likely to have elevated CRP levels, we did see that the proportion of patients with secondary infection is the highest in the underweight group (36.36%), followed by obese (18.75%) and overweight patients (18.52%), and the normal-weight group is the lowest (11.61%; *P* = 0.116). Therefore, we further used multivariable logistic regression to analyze it and found that after adjusting for potential confounders, underweight patients had 3.255-fold higher odds of developing a secondary infection than normal-weight patients (*P* = 0.041). While there was no such phenomenon in the obese group (*P* = 0.157; Table 2). Besides, as BMI increases, more patients had bilateral multiple mottling and ground glass opacities in the lungs (36.36%, 43.75%, 48.15%, and 87.50%, *P* = 0.007). As for the severity of the disease, obese and underweight patients were more likely to develop ALI than normal-weight and overweight patients (87.5, 72.73 vs. 42.86%, 48.15%, *P* = 0.003). Obese and overweight patients were more likely to require human immunoglobulin + systematic corticosteroids therapy than the other two groups (43.75, 35.19 vs. 18.75%, 18.18%, 134, *P* = 0.036).

**Table 2:** Association between body mass index (BMI) and secondary infection on 193 patients with COVID-19.

**BMI, Kg/m^2^**	**N (% Secondary infection)**	**Age adjusted model**	***P*** **-value**	**Multivariable model^†^**	***P*** **-value**
		**ORs (95% CIs)**		**ORs (95% CIs)**	
**Total**					
18.5–23.9	112 (11.61)	1.00		1.00	
24.9–27.9	54 (18.52)	1.663 (0.673–4.110)	0.270	1.912 (0.716–5.105)	0.196
18.5-	11 (36.36)	4.622 (1.167–18.303)	0.029	4.255 (1.060–17.079)	0.041
28+	16 (18.75)	1.689 (0.422–6.770)	0.459	3.058 (0.651–14.361)	0.157

† *Adjusted for age, sex, disease history (hypertension, diabetes, cardiovascular disease and cancer), as appropriate*.

However, there were no significant differences in other clinical features, such as other comorbidities (*P* from 0.236 to 0.824), other symptoms (*P* from 0.485 to 0.901), disease progression characteristics (*P* from 0.072 to 0.500), ARDS (*P* = 0.478), and combination of antibiotics and oxygen therapy (*P* = 0.343).

Table 3 shows that after adjusting for age, sex, comorbidity in all patients, compared to the normal-weight group, the underweight group showed 6.483-fold higher odds (*P* = 0.012), and the obesity group showed 596.5% of higher odds of developing ALI (*P* = 0.022).

**Table 3:** Association between body mass index (BMI) and acute lung injury on 193 patients with COVID-19.

**BMI, Kg/m^2^**	**N (% Acute lung injury)**	**Age adjusted model**	***P*** **-value**	**Multivariable model^†^**	***P*** **-value**
		**ORs (95% CIs)**		**ORs (95% CIs)**	
**Total**					
18.5–23.9	112 (42.86)	1.00		1.00	
24.9–27.9	54 (48.15)	1.064 (0.493–2.297)	0.875	0.809 (0.348–1.882)	0.623
18.5-	8 (72.73)	6.149 (1.325–28.545)	0.020	7.483 (1.568–35.710)	0.012
28+	16 (87.5)	9.509 (1.938–46.660)	0.006	6.965 (1.329–36.495)	0.022

† *Adjusted for age, sex, disease history (hypertension, diabetes, cardiovascular disease and cancer), as appropriate*.

## Discussion/Conclusion

In this study, we included patients in general isolation wards, only two of whom died at the end, one was later transferred to ICU after worsening of the disease and died at the end, the other patient died in the general isolation ward. Other Patients recovered and were discharged from the hospital between January 21, 2020, and April 15, 2020. Some previous studies have found that obesity is positively correlated with the severity of COVID-19. But all of them focused on ICU admission or mortality. So, we wanted to see if the severity of pneumonia in the general ward was also correlated with obesity. In addition to obesity, is underweight correlated with the severity of pneumonia? We found a strong positive correlation between obesity/underweight and ALI in patients with COVID-19. Among the patients in the general isolation wards of our hospital, included in this study 5.70% of patients were underweight, 27.98% of patients were overweight, and 8.29% were obese, respectively. Our research showed that both underweight and obesity significantly increased the risk of developing ALI in patients with COVID-19 (*P* = 0.003). The proportion of patients with bilateral multiple mottling and ground glass opacity in the lungs was highest in the obese group (87.50%), while the underweight group had the lowest proportion (36.36%; *P* = 0.008). In terms of treatment regimens, obese patients had the highest rates of human immunoglobulin + systematic corticosteroids therapy (43.75%) and lowest rates of underweight patients (18.18%, *P* = 0.036). However, underweight patients were more likely to have headache (*P* = 0.029) and develop a secondary infection (*P* = 0.041). The results of multivariable logistic regression analysis in Table 3 also show that compared to the normal-weight group, underweight and obese patients were more likely to develop SARS-CoV-2-induced ALI (*P* = 0.012, *P* = 0.022 respectively). Interestingly, CRP is one of the most typical markers of infection, the proportion of people with elevated CRP in the underweight group was the lowest (18.18%), and that in the obesity group was the highest (62.5%). However, the incidence of secondary infection was the highest in the underweight group (36.36%), followed by the obesity group (18.75%). The probability of secondary infection was 3.255 times higher in the underweight group than in the normal-weight group (*P* = 0.041), while there was no statistical significance in the incidence of secondary infection between the obese and normal-weight groups (*P* = 0.157). These results suggest that in addition to obesity, being underweight is also positively associated with the severity of COVID-19, at least in the general isolation ward. Is this association related to secondary infection? Further research is needed.

In addition, we also found some tendentious results, which may be because our sample size is relatively small, and there are no statistically significant results. Such as the median age of patients in each group increased with increasing BMI (36, 37.5, 40.5, and 44, *P* = 0.176). As BMI increased, the proportion of patients who felt fatigued was increased (36.36, 42.86, 59.26, and 62.50%, *P* = 0.122). Lower albumin and prealbumin levels were more common in underweight and obese patients than in normal and overweight patients (18.18, 18.75 vs. 14.29%, 11.11%; *P* = 0.726 and 60, 50 vs. 38.24%, 36.54%; *P* = 0.421; out of these patients 13 lacked prealbumin values). In terms of treatment, the underweight group had the highest rates of patients who received antibiotic combination treatment, followed by the obese group, overweight group, and normal-weight group (72.73, 68.75, 51.85, and 48.21%, *P* = 0.234). This is consistent with the highest secondary infection rate in underweight patients. The above tedious results require us to expand the sample size to further explore whether there is some definite association between these indicators and BMI. However, no significant differences were found in terms of other comorbidities (*P* = 0.236–0.824), other symptoms (*P* = 0.485–0.901), other disease progression characteristics (*P* = 0.189–0.536), ARDS (*P* = 0.478), and oxygen therapy (*P* = 0.343), etc.

As the COVID-19 pandemic is unfolding around the world (8). Increasing evidence indicates that obesity is an independent risk factor for severe illness and death from COVID-19 (1, 2, 9–13). There is an urgent need to further clarify the relationship between BMI and the severity of COVID-19 (14). The prevalence of obesity has been increasing over several decades, it is defined as “abnormal or excessive fat accumulation that may impair health” and is categorized by BMI (kg/m^2^) (15–17). In fact, obesity is much more than simply a state of mass loading (18), it is the most common chronic metabolic disease in the world. Obesity can affect almost every organ and tissue of the body (15). The obese state is associated with changes in the gut microbiome, endogenous hormone levels, cellular metabolism, lipid handling, immune function, insulin resistance, thrombosis, pharmacokinetic and pharmacodynamic properties of drugs, inflammatory, neurologic, dietary factors, and circulating factors (8, 18). These factors have multiple detrimental effects on the respiratory system, and fundamentally alter the pathogenesis and pathophysiology of lung health and diseases (18). In addition, obesity can cause impaired pulmonary endothelial cell functions and respiratory mechanics, increased airway resistance, impaired gas exchange, and other pathophysiological characteristics, such as decreased respiratory muscle strength and lung volumes, and even obstructive sleep apnoea (19–21). So, obesity is a major risk factor for the development of a wide range of respiratory diseases (15, 18), and it will increase the prevalence and morbidity of lung diseases (15). In addition, adipose tissue may serve as a reservoir for SARS-CoV2 owing to its high levels of expression of angiotensin-converting enzyme-2 (ACE-2), the transmembrane enzyme that SARS-CoV-2 uses for cell entry (22, 23). Asians often display lower cardiorespiratory fitness and carry proportionally more fat tissue at lower BMIs, so the dangers of obesity are even more pronounced in Asians (8). In our study, we found that both underweight and obese patients were more likely to develop ALI. But there were some differences between the obese and underweight groups, including the fact that the obesity group had the highest percentage of patients with lower-than-normal lymphocyte counts. The obese group had the highest and the underweight group had the lowest number of patients with elevated CRP value, bilateral multiple mottling, and ground glass opacity on lung CT scans. Underweight patients were more likely to have a headache and develop a secondary infection. This seems to suggest that both obesity and underweight are risk factors for pneumonia progression, but they differ in their disease-related mechanisms. Previous studies on COVID-19 only paid attention to the harm of obesity, but we found that in addition to obesity, the incidence of ALI in underweight patients was also increased, even higher than the obese patients. Although CRP levels did not tend to be elevated, the underweight group was significantly more likely to develop secondary infections than the other groups. Our findings are consistent with some earlier studies, one of the studies showed that underweight and obese were both independent risk factors for Primary Graft Dysfunction (PGD) and early mortality after lung transplantation (24–26). Both underweight and obesity may increase the risk of infection-related deaths (25). The poor prognosis among underweight patients may be related to a reduced capacity to tolerate surgery because of their underlying malnutrition and skeletal muscle mass loss, termed sarcopenia (27, 28). In several other studies, low BMI was an independent risk factor of morbidity and mortality in patients who underwent pulmonary lung resection (29, 30), and the incidence of cerebrovascular and pulmonary complications tended to be higher in patients with low BMI than in other even obese patients (31). Besides, being underweight is a risk factor for death among patients with chronic obstructive pulmonary disease (COPD) (32). Earlier studies found that death among underweight patients with COPD might be attributable to diaphragmatic weakness and respiratory failure related to a poor nutritional status (33). In adults with COVID, metapneumovirus, parainfluenza, and rhinovirus, underweight and morbidly obese participants had more severe diseases than normal-weight participants. Participants that were underweight (OR: 4.07) and morbidly obese (OR: 2.78) were more likely to be hospitalized as compared to normal-weight adults (34). It is worth noting that in our study hypoalbuminemia, which represents low nutrient levels, was slightly more prevalent in the underweight and obese groups than in normal and overweight patients (18.18%, 18.75% vs. 14.29%, 11.11%; *P* = 0.726) and the prealbumin, which is more sensitive to malnutrition than albumin, its lower than normal level was moderately more prevalent in the underweight and obese patients than in normal and overweight patients (60%, 50% vs. 38.24%, 36.54%; *P* = 0.421). But none of them reached statistical significance. Previous studies have shown that albumin encompasses anti-inflammatory, antioxidant, and anticoagulant properties (35, 36). Hypoalbuminemia is a feature of acute and chronic inflammation (35). COVID-19 displays low levels of human serum albumin (HSA) which were independently associated with an increased risk of thrombotic events and mortality (35–38). Besides, low binding of nutrients in the HSA pool may be a contributor to severe COVID-19 at the systemic stage (39). The fact that underweight or obese individuals are at high risk of suffering severe cases of COVID-19 may be related to their hypoalbuminemia. Therefore, the results we got above do not seem to be a coincidence. Perhaps due to the small sample size, it did not reach statistical significance. Therefore, expanding the sample size is the next step of our research. Previous studies on COVID-19 focused on obese patients and noted an association between severity of illness and obesity. However, we have shown that the risk actually follows a “U”-shaped curve based on BMI level, where patients at both extremes (i.e., those that are underweight or obese) were more likely to develop ALI when compared with normal-weight patients. Our study had some limitations, including a small sample size and being limited by being retrospective and from a single institution. And because most of them are medical workers, the patients are mostly women. No smoking history was included, and no children were included.

For a more detailed investigation regarding the effect of underweight conditions on the severity of COVID-19, a broader analysis should be considered. Due to the small sample size and more than half of the patients were nonemergency (mild or common type) patients with COVID-19, the number of ARDS patients is smaller, resulting in no statistically significant difference in the number of patients with ARDS between groups. In addition, since BMI can be evaluated easily, we only assessed BMI, albumin and prealbumin, we may additionally need to estimate other biomarkers indicative of the nutritional status and muscle mass. Obesity is a huge health and economic burden (15). Weight loss can reverse many of these problems (15). Health care planners should consider the impact of obesity on future respiratory health resources and provide appropriate recommendations for weight loss to promote healthy weight in patients with lung diseases (15, 25). But thinner is not always better, and maintaining a normal weight is essential to stay healthy, especially for those patients with SARS-Cov-2 infection. Besides, obese patients are generally in economically developed areas where medical conditions are also relatively good and can be effectively treated. However, underweight patients are more common in poor economic areas, and these patients are difficult to enjoy timely and appropriate treatment. Even if the vaccine is available, they are unlikely to be able to afford the cost of vaccinations. Therefore, we believe that underweight patients are more likely to be victims of COVID-19 than obese patients. While previous studies have focused on obesity as a risk factor for severe disease, we believe that perhaps underweight patients need more attention from us. In conclusion, our findings suggest that adults, who are underweight or obese, may be at increased risk of developing severe disease due to SARS-Cov-2 infections. Clinicians should keep the BMI of a patient in mind when evaluating risk and deciding on a course of treatment.

## Data Availability Statement

The original contributions presented in the study are included in the article/supplementary material, further inquiries can be directed to the corresponding author/s.

## Ethics Statement

The studies involving human participants were reviewed and approved by the ethics committee of Tongji Medical College, Huazhong University of Science and Technology. The ethics committee waived the requirement of written informed consent for participation.

## Author Contributions

PY designed the study, performed statistical analyses, and drafted the manuscript. RP, LL, and H-RL contributed to data collection and interpretation. S-LL contributed to data interpretation and critical revision of the manuscript. PY and LZ take responsibility for the integrity of the work as a whole, from inception to published article. All authors contributed to the article and approved the submitted version.

## Conflict of Interest

The authors declare that the research was conducted in the absence of any commercial or financial relationships that could be construed as a potential conflict of interest.

## Publisher's Note

All claims expressed in this article are solely those of the authors and do not necessarily represent those of their affiliated organizations, or those of the publisher, the editors and the reviewers. Any product that may be evaluated in this article, or claim that may be made by its manufacturer, is not guaranteed or endorsed by the publisher.
